# Effects of stimulation site and protocol on autonomic responses to auricular vagus nerve stimulation

**DOI:** 10.3389/fnins.2025.1692530

**Published:** 2025-11-28

**Authors:** Alireza Gharabaghi, Marius Keute

**Affiliations:** 1 Institute for Neuromodulation and Neurotechnology, University Hospital and University of Tübingen, Tübingen, Germany; 2 Center for Digital Health, Tübingen, Germany; 3 Center for Bionic Intelligence Tübingen Stuttgart (BITS), Tübingen, Germany; 4 German Center for Mental Health (DZPG), Tübingen, Germany

**Keywords:** transcutaneous auricular vagus nerve stimulation (taVNS), heart rate variability (HRV), standard deviation of heartbeat intervals (SDNN), autonomic modulation, neurovisceral integration, brain-body interaction, control site, stimulation parameters

## Abstract

Transcutaneous auricular vagus nerve stimulation (taVNS) modulates autonomic function, but the influence of stimulation site and protocol remains unclear. In two within-subject experiments in young healthy participants (study 1: *n* = 15, study 2: *n* = 21), we examined how these parameters affect heart rate variability (HRV) as participants performed a visual detection task to stabilize arousal state while randomized stimulation blocks were applied. In study 1, burst (5 pulses at 25 Hz repeated every second), 1 Hz, and 25 Hz protocols were applied at the cymba conchae, with burst stimulation also delivered at the earlobe. In study 2, burst stimulation was applied at cymba conchae, fossa triangularis, tragus, and earlobe sites. While most HRV indices remained unchanged, stimulation-specific effects were observed for SDNN, a global marker of autonomic tone. Burst stimulation at both cymba conchae and earlobe sites produced notable SDNN changes, suggesting sensitivity of cardiac variability to stimulation protocols. These findings indicate that taVNS parameters modulate autonomic output and suggest that earlobe stimulation may evoke measurable autonomic responses under certain conditions. Further studies with larger samples and extended physiological and behavioral monitoring are warranted to confirm these conclusions.

## Introduction

The vagus nerve is a central pathway for brain-body communication, conveying afferent and efferent signals that regulate a wide range of autonomic, cognitive, and affective functions (Neuhuber and Berthoud, 2021; Yuan and Silberstein, 2016). It is a key conduit of interoceptive information, integrating cardiovascular, metabolic, immune, and emotional processes to support adaptive behavior (Karemaker, 2022; Paciorek and Skora, 2020). Transcutaneous auricular vagus nerve stimulation (taVNS), a non-invasive technique targeting the auricular branch of the vagus nerve, has gained traction as both a research tool and therapeutic strategy across a broad range of neurological, psychiatric, inflammatory, cardiological and metabolic conditions (Austelle et al., 2024; Kaniusas et al., 2019). Yet despite its promise, the physiological effects of taVNS remain incompletely understood and often contradictory, both clinically (Yap et al., 2020) and neurophysiologically (Schuerman et al., 2021).

Recent work has raised questions about the consistency and directionality of taVNS effects, challenging the widespread view that taVNS increases vagal tone and heart rate variability (HRV) (Borges et al., 2019; Wolf et al., 2021). Some researchers have proposed that taVNS induced reductions in HRV and that this may reflect increased arousal or central neuromodulatory effects rather than vagal efferent activation (Kaduk et al., 2025). Since physiological responses to taVNS may be influenced by fluctuations in alertness or engagement, several studies have incorporated tasks or stimuli to stabilize the behavioral state during stimulation (Fischer et al., 2018; Wienke et al., 2023). Following this rationale, we delivered stimulation while participants engaged in a continuous visual detection task throughout the experimental session.

Reported discrepancies are further complicated by methodological heterogeneity (Ahmed et al., 2022; Verma et al., 2021). Stimulation site and protocol vary widely across studies (Badran et al., 2018; Thompson et al., 2021). While the cymba conchae and tragus are often targeted based on anatomical innervation, the earlobe is frequently used as a control despite growing evidence that it may produce physiological responses (Butt et al., 2020; Yakunina et al., 2017). Similarly, burst stimulation protocols, which mimic natural vagal firing, may engage vagal afferents more effectively than continuous patterns, yet they have not been consistently applied or compared (D’Agostini et al., 2023; Shen et al., 2022).

In our previous work (Machetanz et al., 2021b), we found that burst stimulation at specific auricular sites (notably cymba conchae and fossa triangularis) increased HRV parameters such as SDNN and RMSSD. However, this study did not include protocol comparisons, nor did it contrast effects at target vs. control sites. Moreover, arousal state was not explicitly managed, leaving open questions about possible confounding influences.

**FIGURE 1:**
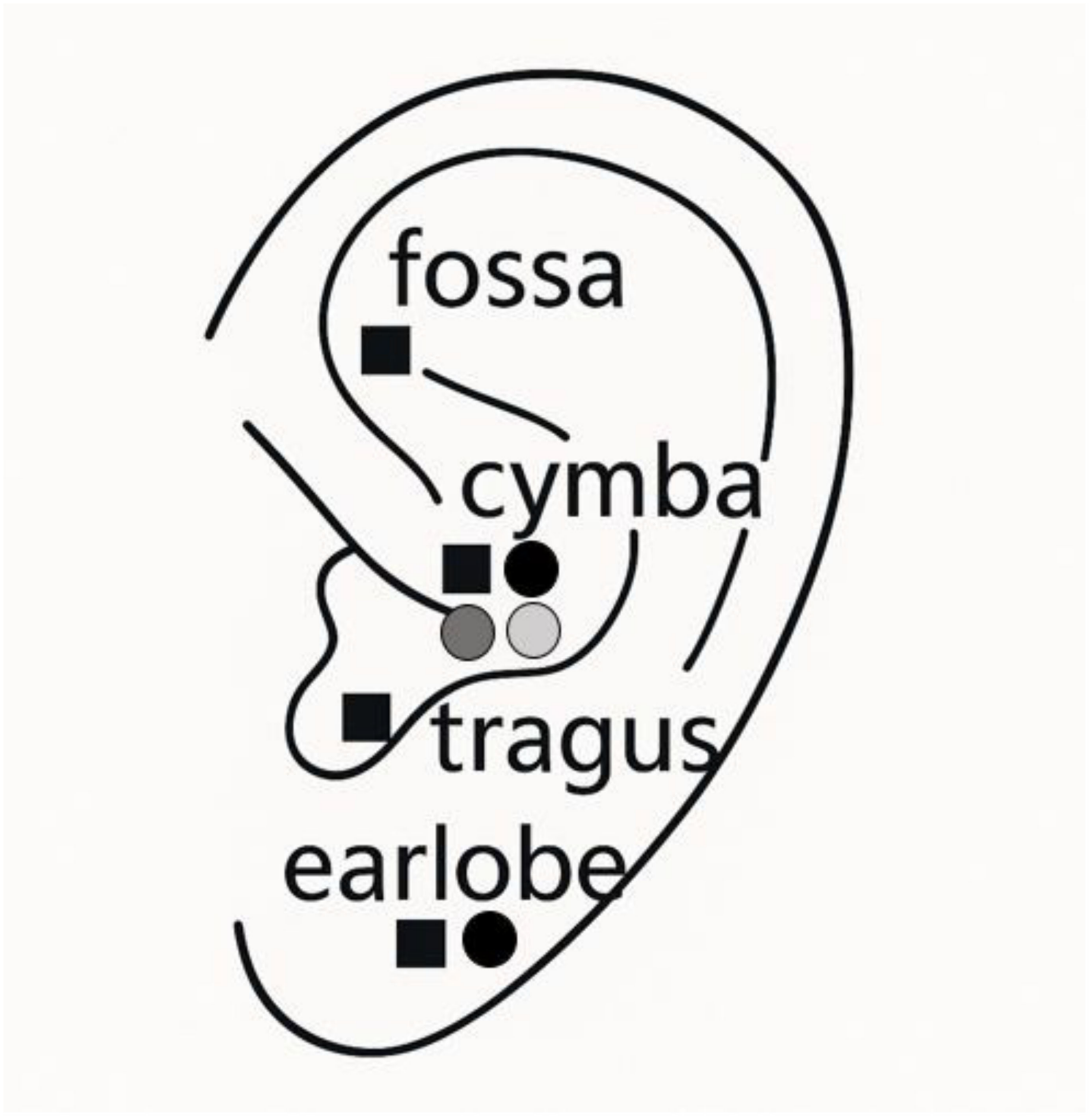
Study design overview with stimulation protocols and auricular sites. Schematic illustration of the left outer ear showing the four auricular locations targeted in two within-subject experiments. Circles denote conditions in the protocol study, where stimulation was applied at the cymba conchae and earlobe: black circles represent burst stimulation, the dark gray circle represents 1 Hz-stimulation (slow), and the light gray circle represents 25 Hz-stimulation (fast). Black squares indicate stimulation sites tested in the site study, where burst stimulation was applied at the fossa, cymba, tragus, and earlobe.

To address these gaps, the present study used a dual-experiment, within-subject design to systematically test the independent contributions of stimulation protocol and site to autonomic outcomes. In the first experiment, participants received continuous 25 Hz, continuous 1 Hz, and burst stimulation (5 pulses at 25 Hz repeated every second) at the cymba conchae, with the burst protocol additionally applied to the earlobe as a comparator condition. In the second experiment, burst stimulation was applied to four sites: cymba, fossa, tragus, and earlobe (Figure 1). Autonomic responses were assessed via heart rate and HRV, focusing on SDNN as an index of overall HRV influenced by both sympathetic and parasympathetic activity rather than vagal tone alone.

Our findings offer new insights into the parameter sensitivity and site specificity of taVNS-induced autonomic modulation, helping to refine current interpretations and inform future applications.

## Methods

### Participants

Two complementary within-subject experiments were conducted with healthy young adults. Exclusion criteria were a history of neurological disorders, cognitive or psychiatric impairments, habitual drug or alcohol consumption, or any other medical condition that could interfere with autonomic or cardiovascular function. All stimulations were applied unilaterally to the left ear for consistency across participants and conditions.

All participants provided informed consent. The study was approved by the Ethics Committee of the Medical Faculty of the University of Tübingen and was conducted in accordance with the Declaration of Helsinki and relevant institutional guidelines.

### Design and procedure

The order of stimulation blocks was randomized across participants. All blocks for each participant were completed within a single experimental session, and sessions were scheduled during daytime. Stimulation was delivered as described previously (Keute et al., 2021; Machetanz et al., 2021a,b) using a programmable current generator (STG 4008, Multi Channel Systems GmbH, Reutlingen, Germany) at an intensity of 2 mA with a pulse width of 200 μs. Waveforms were triggered by a customized Python program and delivered through a bipolar spherical probe (GVB-gelimed GmbH) with a probe diameter of 2 mm and an inter-probe distance of 5 mm. During stimulation, the examiner held the probe continuously at the designated site, resting the arm on an armrest to maintain stable application pressure and consistent current delivery across conditions.

We investigated the conventional 1 and 25 Hz stimulation protocols and additionally applied a biomimetic burst protocol combining these frequencies, consisting of five pulses at 25 Hz repeated every second. This protocol has been used in our previous studies (Keute et al., 2021; Machetanz et al., 2021a,b) and was inspired by experimental work in rabbits showing that intermittent (0.5–1 Hz) rather than continuous (5–25 Hz) vagal stimulation more closely reproduces physiological vagal firing patterns affecting cardiac activity (Iwao et al., 2000).

In study one (protocol comparison), each participant completed four randomized blocks:

1. Slow continuous 1 Hz stimulation at the cymba conchae
2. Fast continuous 25 Hz stimulation at the cymba conchae
3. Burst stimulation at the cymba conchae (5 pulses at 25 Hz repeated every second)
4. Burst stimulation at the earlobe

Each block began with a 5-min resting baseline period, followed by three consecutive 5-min stimulation epochs. Short participant-controlled breaks separated the blocks to minimize fatigue and maintain alertness.

In study two (site comparison), each participant completed four randomized blocks of burst stimulation applied to different auricular sites:

1. Cymba conchae
2. Fossa triangularis
3. Tragus
4. Earlobe

Burst stimulation consisted of five pulses at 25 Hz, repeated every second. As in study one, each block began with a 5-min resting baseline period followed by three consecutive 5-min stimulation epochs, with short participant-controlled breaks in between.

### Task

To maintain a stable arousal state during stimulation, participants performed a continuous visual detection task throughout each session. A white fixation cross was presented at the center of the screen. At pseudorandom intervals, one of the four arms of the cross briefly lengthened (100 ms), and participants indicated the elongated arm using the corresponding arrow key. Visual error feedback was provided for incorrect or delayed responses (>2 s). This task helped minimize spontaneous fluctuations in alertness.

### Physiological recording and analysis

Cardiac activity was recorded continuously using two-lead chest electrocardiography (ECG). Data were analyzed using Python 3 and the biosppy package. After automated R-peak detection, the following standard indices of heart rate variability (HRV) were extracted: Mean heart rate; PNN50 (proportion of successive heartbeat intervals differing by >50 ms); RMSSD (root mean square of successive heartbeat interval differences); SDNN (standard deviation of heartbeat intervals). All ECG recordings were visually inspected to verify correct R-peak detection and signal quality. No data were missing or excluded. For each condition, HRV indices were computed separately for the three consecutive 5-min stimulation epochs. These values were then averaged using the arithmetic mean to yield a single representative measure per condition for each participant to provide a more robust estimate of autonomic activity and to reduce the influence of transient physiological fluctuations. Frequency-domain HRV parameters (LF, HF) were not included because individual 5-min stimulation windows are too short for reliable spectral estimation and are highly sensitive to noise.

### Statistical analyses

Due to the small sample sizes and non-normal distribution of the HRV data, we used non-parametric statistics:

1. Within-condition comparisons (baseline vs. stimulation epochs) were performed using Wilcoxon signed-rank tests.
2. Between-condition comparisons (across protocols or stimulation sites) were assessed using Friedman tests.

## Results

### Heart rate variability responses to taVNS

Across both experiments, stimulation-specific effects on heart rate variability (HRV) were observed. Among all extracted HRV measures (SDNN, RMSSD, PNN50, and mean heart rate), only SDNN exhibited significant changes in response to certain stimulation conditions. RMSSD, PNN50, and mean heart rate showed no significant effects across any protocol or stimulation site (all *p* > 0.10), indicating that the observed effects were specific to SDNN, a global marker of autonomic tone that integrates both sympathetic and parasympathetic influences.

### Study one: protocol comparison

**FIGURE 2:**
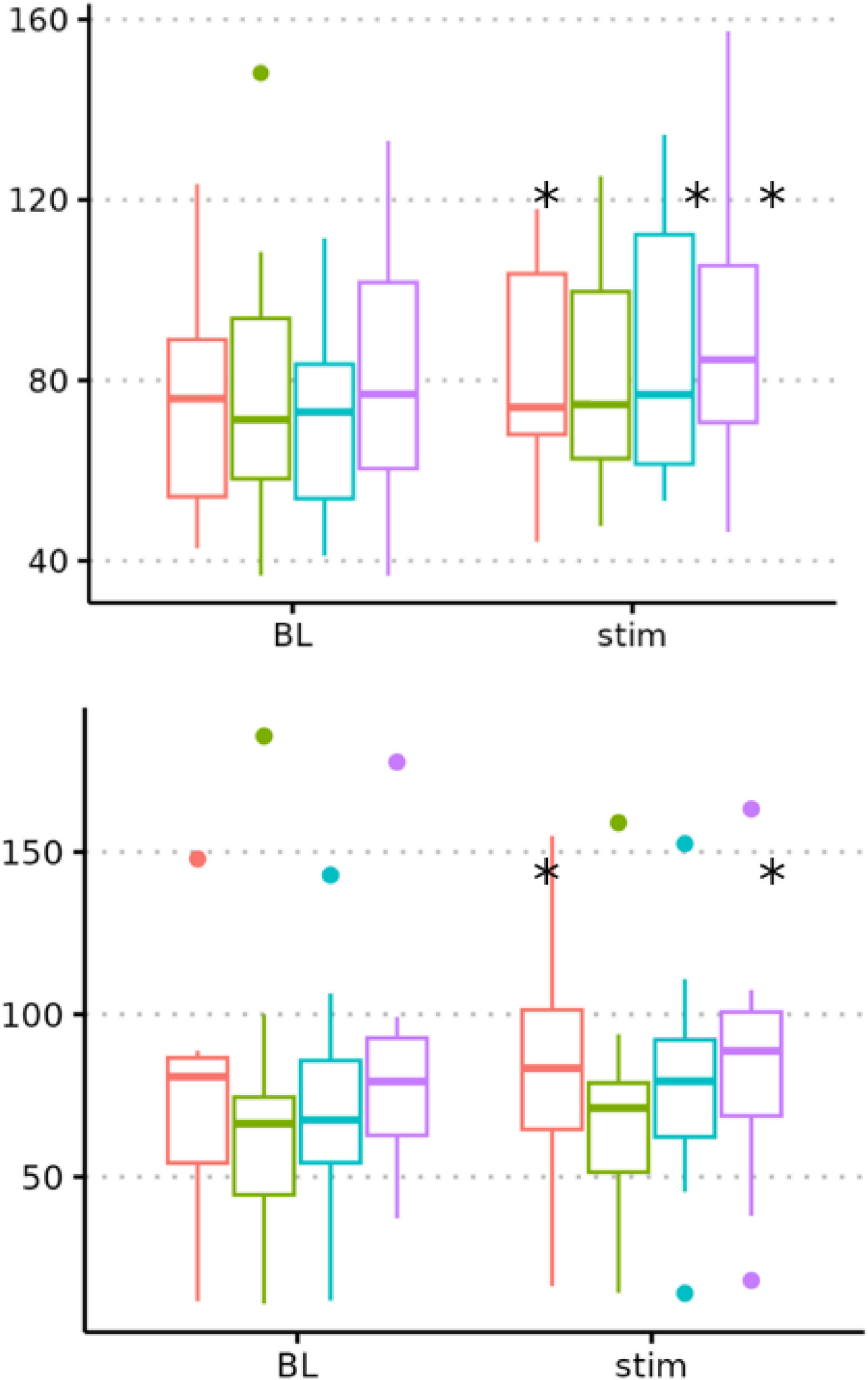
Standard deviation of heartbeat intervals (SDNN) responses to taVNS across stimulation protocols **(A)** and auricular sites **(B)**. Boxplots show median values and interquartile ranges for SDNN (ms) at baseline and during the averaged stimulation period. In the top panel (**A**, protocol comparison), different stimulation protocols – burst (red), fast (green), and slow (purple) – were applied to the cymba conchae, along with a burst protocol at the earlobe (cyan). In the bottom panel (**B**, site comparison), different stimulation sites – cymba (red), fossa (green), tragus (purple), and earlobe (cyan) – were compared using the same burst protocol. Significant (*) within-condition differences between baseline (BL) and stimulation (*p* < 0.05) were observed for burst cymba, slow cymba, and burst earlobe stimulation in the protocol comparison, and for burst cymba and burst earlobe stimulation in the site comparison. No significant differences between protocols or between sites were found.

In the protocol study (*n* = 15; 3 females; age range 20–30 years; mean age = 24.8 years), SDNN changed significantly from baseline to stimulation during burst cymba, slow cymba, and burst earlobe stimulation, whereas fast cymba stimulation produced no significant change (Figure 2). Median and interquartile range (IQR) values for SDNN at baseline and during stimulation, along with Wilcoxon test results, chi-square statistics, and effect sizes (r), are presented in Table 1. Between-protocol comparisons using a Friedman test revealed no significant overall effect across stimulation protocols (χ^2^ = 3.17, *p* = 0.225).

**TABLE 1:** Standard deviation of heartbeat intervals (SDNN) values (median [IQR]) and statistical results for baseline–stimulation comparisons.

Experiment	Condition	*n*	Baseline median (IQR)	Stimulation median (IQR)	*P*-value	χ^2^ (df = 1)	Effect size r	Interpretation
Exp. 1	Burst cymba	15	75 (40–90)	80 (60–100)	0.035	3.38	0.475	Medium
Exp. 1	Slow cymba	15	80 (65–95)	85 (70–110)	0.048	3.05	0.451	Medium
Exp. 1	Fast cymba	15	70 (45–90)	70 (55–95)	0.207	0.66	0.210	Small
Exp. 1	Burst earlobe	15	70 (55–95)	80 (60–100)	0.002	9.55	0.798	Large
Exp. 2	Burst cymba	21	75 (55–90)	85 (60–110)	0.016	5.78	0.525	Large
Exp. 2	Burst fossa	21	65 (45–80)	70 (55–90)	0.380	0.25	0.109	Small
Exp. 2	Burst tragus	21	75 (60–95)	85 (65–105)	0.469	0.14	0.082	Small
Exp. 2	Burst earlobe	21	70 (55–90)	80 (60–100)	0.042	3.26	0.394	Medium

### Study two: site comparison

In the site study (*n* = 21; 11 females; age range 20–30 years; mean age = 25.9 years), significant SDNN changes from baseline to stimulation were found for burst cymba and burst earlobe stimulation, while burst fossa and burst tragus stimulation showed no significant effects (Figure 2). The descriptive and inferential results for all stimulation sites are summarized in Table 1. Between-site comparisons using a Friedman test revealed no significant overall effect (χ^2^ = 2.03, *p* = 0.593).

## Discussion

In this study, we examined how stimulation site and protocol influence autonomic responses to taVNS, using HRV as an index of autonomic function. Among the extracted HRV measures, stimulation-related differences were observed only for SDNN, a global marker of autonomic tone. These effects occurred during burst stimulation at both the cymba conchae and the earlobe.

Our findings add to a growing body of evidence indicating that subtle variations in taVNS parameters can shape physiological outcomes (Badran et al., 2018; Thompson et al., 2021). The present SDNN findings, particularly with burst stimulation, suggest that stimulation patterning may influence global autonomic variability. This likely reflects a more complex interplay between sympathetic and parasympathetic influences rather than a unidirectional shift in vagal tone. The effect may also have been facilitated by the continuous detection task performed during stimulation, which likely elevated baseline arousal levels. As the starting arousal state may modulate the direction and magnitude of taVNS-induced HRV changes, future studies are needed to systematically test how behavioral context interacts with stimulation parameters to shape autonomic outcomes.

Notably, burst stimulation, which mimics the phasic firing properties of natural vagal afferents, produced effects on SDNN. This is consistent with previous work indicating that biomimetic stimulation patterns may better engage brainstem autonomic circuits compared to continuous stimulation (Wienke et al., 2023; Yap et al., 2020). The absence of significant HRV changes with fast continuous stimulation supports the potential importance of stimulation patterning over frequency alone.

Our results also have important implications for the choice of stimulation site. While the cymba conchae is anatomically favored due to its dense vagal innervation (Peuker and Filler, 2002), the earlobe, frequently used as a control site (Yakunina et al., 2017), yielded SDNN changes comparable in magnitude to those observed at the cymba. This challenges the widespread assumption that earlobe stimulation is physiologically inert, indicating instead that it can produce measurable autonomic changes under specific protocols such as burst stimulation, although the underlying neural mechanisms remain unclear and may involve mechanoreceptive or non-vagal afferents, thereby highlighting the need for carefully validated control conditions in taVNS research.

Our results should also be considered in the context of recent systematic reviews and meta-analyses. A Bayesian meta-analysis found no consistent overall effect of taVNS on vagally mediated HRV, highlighting considerable heterogeneity across studies and pointing to stimulation site and protocol as key moderators (Wolf et al., 2021). Similarly, a systematic review of HRV and baroreflex sensitivity in healthy subjects concluded that findings remain inconsistent and emphasized the need for parameter-specific investigations (Soltani et al., 2023). In addition, a systematic review and meta-analysis of randomized controlled trials on cardiovascular effects reported mixed outcomes for HR and HRV and underlined the importance of standardized methods and careful selection of control sites (Hua et al., 2023). Our findings, showing that burst stimulation produced SDNN changes at both cymba conchae and earlobe sites, directly contribute to these identified gaps by demonstrating protocol- and site-dependent effects.

A strength of our study is our attempt to stabilize the behavioral state during stimulation. Because spontaneous fluctuations in arousal can influence HRV independently of direct vagal activation (Shaffer and Ginsberg, 2017), participants performed a continuous visual detection task throughout stimulation. Although task performance metrics (e.g., reaction times, error rates) were not recorded, structured engagement likely reduced state-dependent variability and contributed to the observed effects.

Several limitations should be considered, which may inform the design of future research: First, the moderate sample sizes limited statistical power and precluded detection of interaction effects between stimulation protocol, site, and time. Because this exploratory study aimed to provide initial data rather than test a single confirmatory hypothesis, no formal *a priori* power analysis was conducted. To quantify the magnitude of the observed within-subject effects, effect sizes are reported in Table 1. These values facilitate interpretation beyond significance testing but do not eliminate the increased risk of Type I and Type II errors inherent in small samples. Future studies should use power calculations based on the present effect sizes and apply repeated-measures ANOVA or mixed-effects models to better characterize these interactions. The current samples were also too small to reliably examine potential sex-related differences in autonomic responses, which should be addressed systematically in larger cohorts.

Second, because testing sessions were scheduled at varying times of day, circadian influences on autonomic function may have contributed to between-subject variability. Third, only time-domain HRV indices were analyzed; frequency-domain measures (LF, HF) were excluded because the applied stimulation windows provide insufficiently reliable spectral estimates. Alternative analytic approaches suitable for short recording segments, such as detrended fluctuation analysis and point-process modeling, have recently been applied in taVNS research (Le Roy et al., 2023; Sclocco et al., 2019; Toschi et al., 2023) and may complement conventional time-domain indices in future studies. Forth, HRV values from the three consecutive stimulation epochs were averaged to obtain more stable condition-level estimates, which may obscure temporal dynamics within each stimulation period. Fifth, the experimental protocol did not include a pre-task resting baseline or post-stimulation recovery recording, which limits interpretation of absolute autonomic shifts relative to task-related arousal. In addition, the lack of behavioral performance data such as reaction times or error rates prevents empirical confirmation that the continuous detection task successfully stabilized arousal. Future studies should incorporate both behavioral and physiological indices to validate task-related arousal stability.

Finally, stimulation intensity, waveform configuration, and duty cycle were not systematically varied, leaving open questions about the optimal parameter space for taVNS. Future studies should explore this parameter space more comprehensively and include direct comparisons of control sites to clarify their physiological relevance. Concurrent monitoring of additional behavioral and neurophysiological measures (e.g., pupillometry, EEG, respiration) may help distinguish arousal-driven from vagus-specific effects and clarify interactions between cortical and autonomic processes (Keute et al., 2021; Machetanz et al., 2021a).

In conclusion, these findings suggest that both stimulation site and protocol influence autonomic responses to taVNS. Burst stimulation at the cymba conchae and the earlobe was associated with SDNN changes, indicating that specific stimulation configurations can modulate global autonomic variability. These insights have implications for optimizing taVNS applications in experimental and clinical contexts and underscore the importance of carefully selecting stimulation parameters and control conditions in future research.

## Data Availability

The raw data supporting the conclusions of this article will be made available by the authors, without undue reservation.
